# Identification of Key LncRNAs and Pathways in Prediabetes and Type 2 Diabetes Mellitus for Hypertriglyceridemia Patients Based on Weighted Gene Co-Expression Network Analysis

**DOI:** 10.3389/fendo.2021.800123

**Published:** 2022-01-24

**Authors:** Shoumeng Yan, Mengzi Sun, Lichao Gao, Nan Yao, Tianyu Feng, Yixue Yang, Xiaotong Li, Wenyu Hu, Weiwei Cui, Bo Li

**Affiliations:** ^1^ Department of Epidemiology and Biostatistics, School of Public Health, Jilin University, Changchun, China; ^2^ Department of Endocrinology, The First Hospital of Jilin University, Changchun, China; ^3^ Department of Nutrition and Food Hygiene, School of Public Health, Jilin University, Changchun, China

**Keywords:** diabetes, lncRNAs, qRT-PCR, WGCNA, hypertriglyceridemia

## Abstract

**Aims:**

Prevalence of prediabetes and type 2 diabetes mellitus(T2DM) are increasing worldwide. Key lncRNAs were detected to provide a reference for searching potential biomarkers of prediabetes and T2DM in hypertriglyceridemia patients.

**Methods:**

The study included 18 hypertriglyceridemia patients: 6 newly diagnosed type 2 diabetes patients, 6 samples with prediabetes and 6 samples with normal blood glucose. Weighted gene co-expression network analysis (WGCNA) was conducted to construct co‐expression network and obtain modules related to blood glucose, thus detecting key lncRNAs.

**Results:**

The green, yellow and yellow module was significantly related to blood glucose in T2DM versus normal controls, T2DM versus prediabetes, prediabetes versus normal controls, respectively. ENST00000503273, ENST00000462720, ENST00000480633 and ENST00000485392 were detected as key lncRNAs for the above three groups, respectively.

**Conclusions:**

For hypertriglyceridemia patients with different blood glucose levels, ENST00000503273, ENST00000462720 and ENST00000480633 could be potential biomarkers of T2DM.

## Introduction

Hyperglycemia promotes a variety of reactions, including oxidative stress and the formation of advanced glycosylated end products, which have been associated with structural and functional changes in blood vessels that eventually cause dysfunction of several organs, especially the heart, nerves, eyes, and kidneys (1). Prevalence of prediabetes is increasing worldwide and more than 470 million people will have prediabetes by 2030 (2). Previous studies indicated that prediabetes was associated with an increased risk of coronary heart disease, stroke, and all-cause mortality (3). Meanwhile, it is estimated that the number of patients with diabetes were expected to increase to 693 million by 2045. Type 2 diabetes mellitus accounts for about 90%-95% of patients with diabetes (4). Compared with people who do not have diabetes, T2DM patients have a 15% increased risk of all-cause mortality (5). Specially, hypertriglyceridemia can reduce peripheral insulin sensitivity. Meanwhile, insulin resistance leads to the occurrence of hypertriglyceridemia. Therefore, a vicious circle is established (6). Furthermore, T2DM patients with hypertriglyceridemia usually have a higher prevalence of diabetic complications (7). Therefore, whether from a public health perspective or a clinical perspective, prediabetes and T2DM patients with high triglycerides should be paid more attention.

Current research is still searching for novel reliable non-invasive biomarkers to replace the invasive sampling methods for the diagnostic protocols (8, 9). Meanwhile, healthy individuals release exosomes with a cargo of different RNA, DNA, and protein contents into the circulation. Therefore, the above molecules can be measured non-invasively as biomarkers of healthy and diseased states (10). Long noncoding RNAs (lncRNAs) represent a class of transcripts longer than 200 nucleotides with limited protein-coding potential (11). Increasing evidence indicated that lncRNAs could interfere with gene expressions and signaling pathways at various stages, play important roles in the regulation of tissue homeostasis and pathophysiological conditions (12). Existing research have indicated that lncRNA were involved in the entire prediabetes biological process (13). Therefore, as a biomarker, lncRNA plays an important role in the prediction and diagnosis of diseases. Li et al. reported that lncRNA ENST00000550337.1 could be a potential diagnostic biomarker for prediabetes (14). In addition, studies have shown that lncRNAs were related to T2DM *via* producing a complex regulatory network through interactions with transcription factors (15). The abnormal expression of lncRNA NONRATT021972 participates in the occurrence and progression of T2DM, in which it was a potential clinical biomarker (16). The emerging evidence indicated that the role of MALAT1 in diabetes complications is both pro-inflammatory and apoptosis in different cell types (17). However, conventional method only focuses on the role of the single gene, the external sample traits cannot be combined (18). Fortunately, weighted gene co-expression network analysis (WGCNA) could solve the problem.

WGCNA was a method of scale-free network analysis proposed by Peter Langfelder and Steve Horvath in 2008. WGCNA can be used for detecting modules of highly correlated genes, and summarizing such modules *via* the module eigengene or an intramodular hub gene, and relating modules to one another and to external sample traits (18). Meanwhile, WGCNA also alleviates the multiple testing problems inherent in microarray data analysis (19). In addition, WGCNA focused on the whole genome information to overview of the signature of gene networks in phenotypes which can avoid bias and subject judgement (20). Based on the above characteristics of WGCNA, we used it to construct a co‐expression network and obtain modules related to blood glucose, thus detecting key genes, and providing a reference for searching potential biomarkers of prediabetes and T2DM in hypertriglyceridemia patients. We present the following article in accordance with the STROBE reporting checklist.

## Materials and Methods

### Participants

The study included 18 hypertriglyceridemia patients: six newly diagnosed type 2 diabetes patients, six samples with prediabetes and six samples with normal blood glucose. All participants were Han Chinese aged 40-65 years and were recruited at the First Hospital of Jilin University from July to September 2020. Patients were diagnosed based on the guidelines for the prevention and control of type 2 diabetes in China (2017 Edition): Patients with type 2 diabetes were defined as fasting plasma glucose (FPG)≥7.0 mmol/L or oral glucose tolerance test (OGTT) two-hour blood glucose ≥11.1 mmol/L. The range of FPG from 6.1-7.0 or the range of OGTT from 7.8-11.1 were regarded as patients with prediabetes. Besides, FPG<6.1 mmol/L and OGTT<7.8 mmol/L could be regarded as the normal controls. Meanwhile, the level of triglycerides (TG) in all participants was >1.7 mmol/L based on the guidelines for prevention and treatment of dyslipidemia in China(2016 Edition). Additionally, all participants had not controlled their blood glucose through drugs or other treatments previously. Meanwhile, all patients with the history of coronary artery disease (CAD), hypertension, atrial fibrillation, myocardial infarction, tumor, acute infectious disease, immune disease, and hematological disease were excluded from the study. All participants have written informed consent and the study was approved by Ethics Committee of the Public Health of the Jilin University, and the privacy of the participants are strictly confidential.

### Blood Sample Collection and RNA Sequencing

For each sample, 9 ml trizol (TAKARA BIO INC., CA, Japan) was added into the whole blood immediately after the blood samples (3 ml) were collected. The ratio of trizol to whole blood is 3:1. And total RNA was isolated and purified using total RNA extraction kit. NanoPhotometer^®^ spectrophotometer (IMPLEN, CA, USA) was used to detect the RNA purity. Meanwhile, RNA integrity was evaluated using the RNA Nano 6000 Assay Kit of the Agilent Bioanalyzer 2100 system (Agilent Technologies, CA, USA). The chain-specific library was constructed by removing the ribosomal RNA. After the library was qualified, Illumina PE150 sequencing was performed according to pooling of the effective concentration of the library and the data output requirements. Followed by the sequencing, we removed reads with adapter and N (N means that the nucleobase information cannot be determined) ≥ 0.002, and low-quality reads from raw data. Meanwhile, Q20, Q30, and GC content were calculated. Finally, we obtained the clean reads. All analyses in the study were based on the clean data.

### Weighted Gene Co-Expression Network Analysis

We used R version 4.0.4 and the package ‘WGCNA’ for data analysis. The WGCNA R software package is a comprehensive collection of R functions for performing various aspects of weighted correlation network analysis (18). We selected the 5000 genes using the Median Absolute Deviation (MAD) algorithm to ensure heterogeneity and accuracy of bioinformatics for co‐expression network analysis (21). Then, to make the constructed network more consistent with the characteristics of scale‐free network and amplify the correlation between genes, an appropriate soft threshold *β* is selected (20, 22). Subsequently, we converted the adjacency matrix into a topological overlap matrix (TOM) to evaluate gene connectivity in the network. Finally, an average linkage hierarchical clustering was performed based on TOM‐based dissimilarity, with a gene dendrogram>30 and cutting height < 0.25 to construct module dendrograms for further analysis (21, 23).

### Screening for Key Modules and Differentially Expressed LncRNAs

The principal component analysis was performed for module eigengenes (ME). Meanwhile, we can assess the relation between MEs and blood glucose and determine the T2DM-related module by combining the clinical data of participants. Meanwhile, the limma R package, based on empirical Bayes methods and linear models, was used to get differentially expressed lncRNAs. The threshold was *P*<0.05 (22).

### Identification of Key lncRNAs and Functional Enrichment Analysis

We take the intersection of all genes in T2DM-related module and differentially expressed lncRNAs to obtain key lncRNAs. Subsequently, these key lncRNAs were mapped to the DAVID (Database for Annotation, Visualization, and Integrated Discovery) dataset (https://david.ncifcrf.gov/) to convert gene symbol to gene ID. Then, we used KOBAS 3.0 (http://kobas.cbi.pku.edu.cn/) to conduct the Functional Enrichment Analysis.

### Quantitative Real‐Time Polymerase Chain Reaction

The blood samples in qRT-PCR experiment were from a cross-sectional survey in Jilin Province. There were 125 T2DM patients and prediabetes who met the inclusion criteria, respectively. Meanwhile, based on the matching of gender and age, we selected the corresponding control blood samples. The total RNA was extracted using the MolPure^®^ Blood RNA Kit (19241ES50, YEASEN) based on the manufacturer’s instructions. Subsequently, we used lnRcute lncRNA First-Strand cDNA Kit (KR202, TIANGEN) to conduct reverse transcription. The cDNA was then analyzed by qRT-PCR using lnRcute lncRNA qPCR Kit (FP402, TIANGEN) on QuantStudio 3 system (Applied Biosystems). The PCR amplification was performed with one cycle at 95°C for 3 min, followed by 40 cycles at 95°C for 5 sec, at 55°C for 10 sec, and at 72°C for 15 sec. The following PCR primers were used: ENST00000503273 primers, forward: 5′- CCTGCCCGCTATGTGACCAATG -3′, reverse: 5′- ACTCCAGCCTGTATCTTCCTCCATC -3′; ENST00000462720 primers, forward: 5′- CTGTGCTTCTGCTTGACTGAGGATC -3′, reverse: 5′-AGGGTGACTGTGAGAGGGTGATG -3′; ENST00000480633 primers, forward: 5′- GAGCCTCGTTCACGGTTCTATGC -3′, reverse: 5′- CAGCCAGCTTGCAGTGACCTTC -3′; ENST00000485392 primers, forward: 5′- TGACGATGAGGTGGCGGTAA -3′, reverse: 5′- GCTCTCGCTGAAACCAGTCC -3′. Expression data were normalized to the expression of β-actin with the 2^−ΔΔCt^ method.

### Statistical Analysis

All statistical analyses were performed by IBM SPSS 24.0 and R version 4.0.4. The package ‘WGCNA’ was used to construct a weighted gene co-expression network. Mean and standard deviation were used to describe the normal continues variables, and the analysis of variance (ANOVA) was performed for the comparison. Meanwhile, median and quartiles were calculated to describe the skewed continues variables, and the Kruskal-Wallis test was used for the comparison. Moreover, Chi-square tests were used to compare categorical variables. A 2-sided *P* value less than 0.05 was considered significant.

## Results

### Basic Situation of the Transcriptome Data and the Determination of Soft Threshold

Basic information of six newly diagnosed type 2 diabetes patients, six samples with prediabetes and six normal controls was shown in **Table 1**. And the transcriptome data were used to perform the study. To ensure the quality of analysis, the cleaned data were used after eliminating low-quality data. The detailed situation was shown in **Table 2**. In order to assess the effect of lncRNA in people with different blood glucose levels, we performed pairwise analysis on the three group: type 2 diabetes versus normal controls, type 2 diabetes versus prediabetes, and prediabetes versus normal controls. Meanwhile, the appropriate soft threshold β were ·determined based on the selected criteria of power value, which was 16, 18 and 16, respectively (**Figures S1–S3**).

**Table 1:** Basic Situation of Participants in the Study.

	T2DM (n=6)	Prediabetes (n=6)	Control (n=6)	*χ^2^/F*	*P*
Gender (male/female)	4/2	4/2	2/4	1.761	0.589
Age (year)	54 ± 6.03	53.17 ± 3.13	52 ± 6.26	0.213	0.811
Weight (kg)	65 (61.25, 75)	65 (62.25, 66.25)	60 (58.75, 77.5)	0.693	0.707
Height (cm)	170.33 ± 5.16	168.67 ± 6.98	164.83 ± 6.80	1.178	0.335
BMI (kg/m^2^)	22.62 ± 2.49	22.84 ± 2.39	24.1 ± 2.7	0.594	0.565
TC (mmol/L)	4.86 ± 0.92	5.19 ± 0.63	5.94 ± 1.2	2.072	0.160
LDL (mmol/L)	2.65 ± 0.52	2.92 ± 0.47	3.52 ± 0.56	4.496	0.030
HDL (mmol/L)	1.09 (0.85, 1.23)	1.04 (0.86, 1.35)	1.31 (1.1, 2.11)	4.060	0.131
TG (mmo/L)	2.59 (2.24, 6.51)	2.5 (1.98, 7.59)	2.29 (1.98, 3.24)	0.947	0.623
Lipoprotein (a) (mg/L)	30.5 (13.5, 213.5)	121 (24.25, 267.5)	66 (36.5, 340.75)	1.263	0.532
Creatinine (μmoI/L)	63.2 ± 7.26	106.5 ± 7.78	68 ± 13.29	13.103	0.002
Uric acid (μmoI/L)	352.5 ± 40	389 ± 65.05	399 ± 116.84	0.314	0.739
ALT (U/L)	18.75 ± 0.96	22.25 ± 5.74	19 ± 4.08	0.906	0.438
AST (U/L)	22 (19, 24.25)	19.5 (12.25, 35)	23 (16.25, 24.5)	0.554	0.758
FPG (mmol/L)	8.8 (7.95, 13.15)	6.55 (6.28, 6.65)	5.25 (4.88, 5.5)	15.205	<0.001
A1C (mmol/mol)	85.8 ± 17.43	46.67 ± 1.53	38.5 ± 2.12	13.081	0.004
A1C (%)	9.96 ± 1.58	6.47 ± 0.15	5.7 ± 0.14	12.832	0.005

**Table 2:** Summary of data from RNA sequencing.

	Raw_reads	Clean_reads	Raw_bases (G)	Clean_bases (G)	Error rate (%)	Q20 (%)	Q30 (%)	GC_content (%)
HTG_D_1	95930240	93380952	14.39	14.01	0.02	98.25	95.03	58.36
HTG_D_2	95047612	93684148	14.26	14.05	0.02	98.08	94.78	60.28
HTG_D_3	87308266	85200132	13.10	12.78	0.02	98.14	94.77	56.51
HTG_D_4	93872544	92111104	14.08	13.82	0.03	97.60	93.34	59.32
HTG_D_5	98171594	95680364	14.73	14.35	0.02	98.03	94.36	59.55
HTG_D_6	92939324	90560052	13.94	13.58	0.03	97.77	93.84	56.14
HTG_P_1	94041630	92351516	14.11	13.85	0.03	97.78	94.36	62.02
HTG_P_2	92027882	90468058	13.80	13.57	0.02	98.29	95.24	59.29
HTG_P_3	93457176	91128506	14.02	13.67	0.02	98.41	95.54	60.52
HTG_P_4	84170598	81790586	12.63	12.27	0.02	97.91	94.53	59.49
HTG_P_5	97877050	95285304	14.68	14.29	0.03	97.47	93.82	63.35
HTG_P_6	93912472	92728294	14.09	13.91	0.02	98.00	94.60	59.82
HTG_N_1	85958252	84363446	12.89	12.65	0.02	98.32	95.41	60.36
HTG_N_2	85005964	83070364	12.75	12.46	0.02	97.83	94.44	61.92
HTG_N_3	91876150	89511812	13.78	13.43	0.02	97.92	94.57	59.15
HTG_N_4	91519536	89611444	13.73	13.44	0.02	98.14	94.97	57.04
HTG_N_5	91153458	89365918	13.67	13.40	0.02	98.20	95.04	58.43
HTG_N_6	84993186	83520980	12.75	12.53	0.02	98.21	95.01	57.19

### Type 2 Diabetes *Versus* Normal Controls

**Figure 1:**
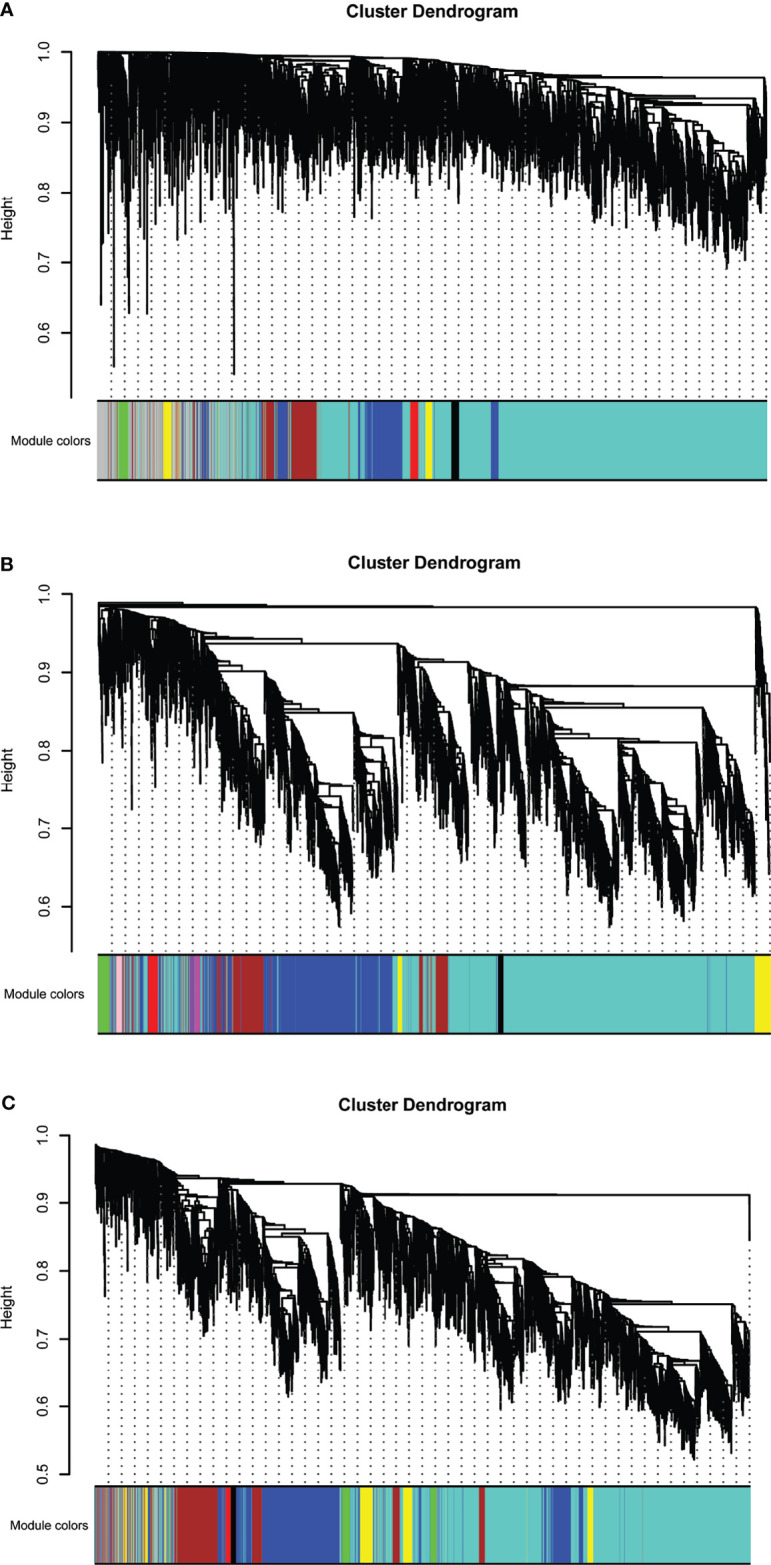
Hierarchical clustering dendrograms of identified co-expressed genes in modules in **(A)** T2DM versus normal controls, **(B)** T2DM versus prediabetes and **(C)** prediabetes versus normal controls (different colors represent different modules).

**Figure 2:**
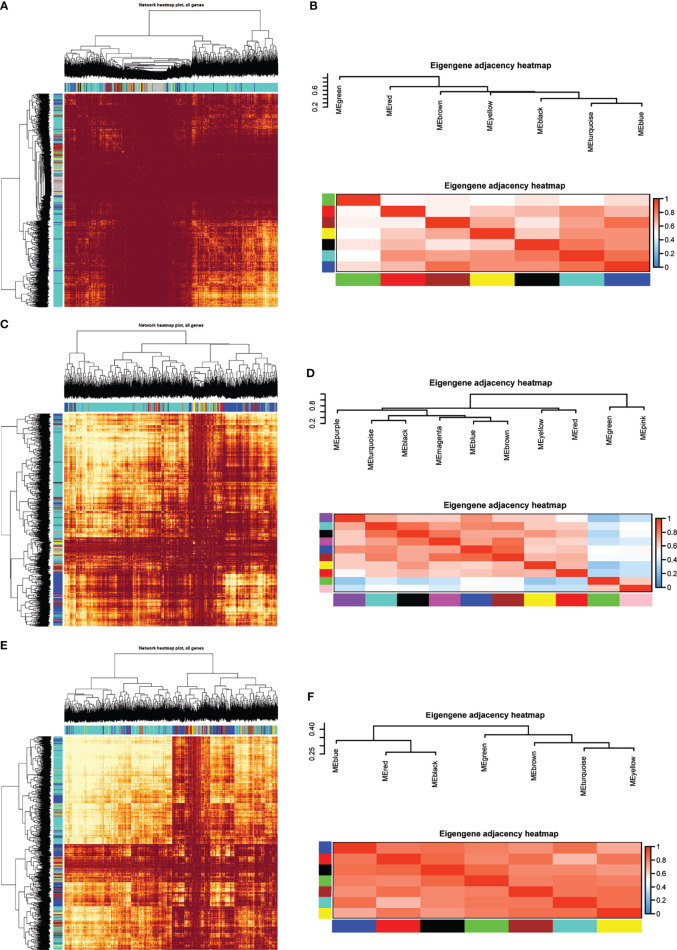
Module preservation analysis. Visualization of the WGCNA network using a heatmap plot in **(A)** T2DM versus normal controls, **(C)** T2DM versus prediabetes and **(E)** prediabetes versus normal controls (The heatmap depicts the topological overlap matrix (TOM) among all genes included in the analysis). **(B, D, F)** represented the heatmap plot of the adjacencies of modules in T2DM versus normal controls, T2DM versus prediabetes and prediabetes versus normal controls, respectively.

**Figure 3:**
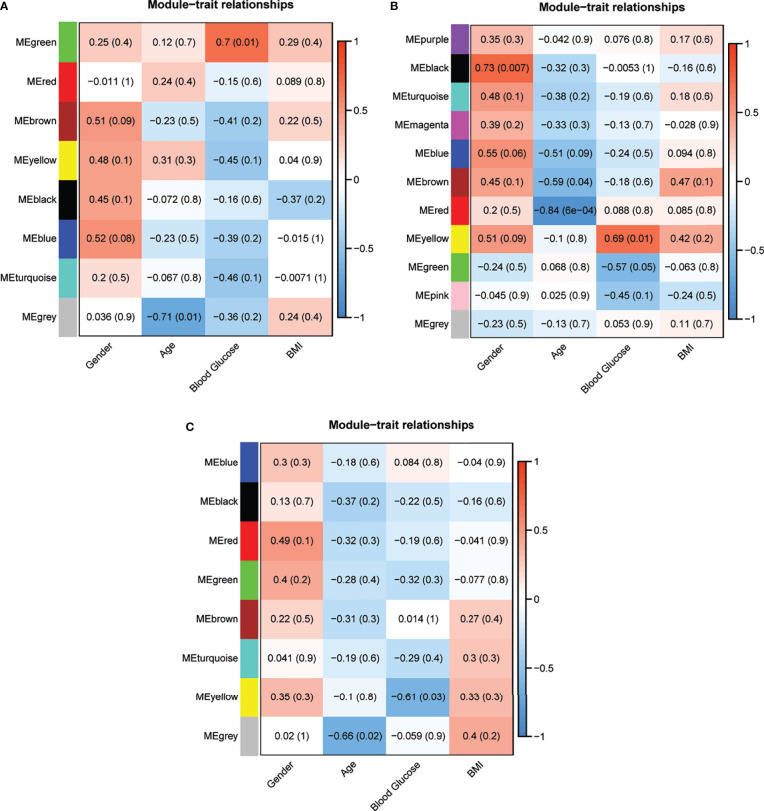
Correlation analysis of the modules and clinical traits in **(A)** T2DM versus normal controls, **(B)** T2DM versus prediabetes and **(C)** prediabetes versus normal controls (Each cell contained the corresponding correlation and P value).

**Figure 4:**
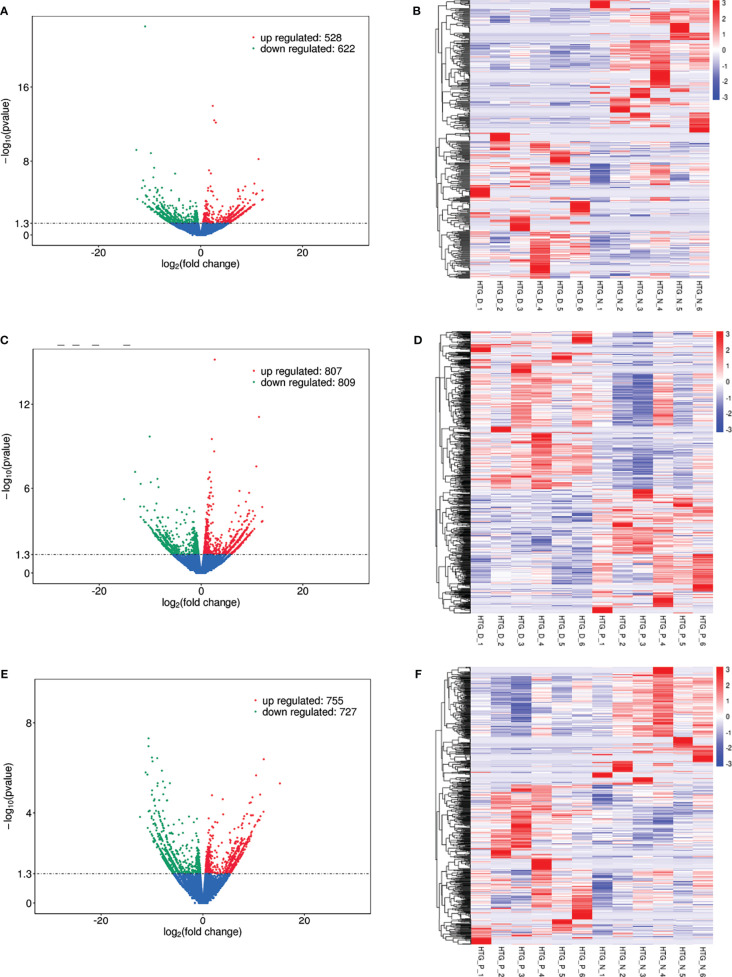
Differentially expressed lncRNAs in **(A)** T2DM versus normal controls, **(C)** T2DM versus prediabetes and **(E)** prediabetes versus normal controls with volcano plot, and **(B, D, F)** represented the heatmap plot for above groups, respectively.

**Figure 5:**
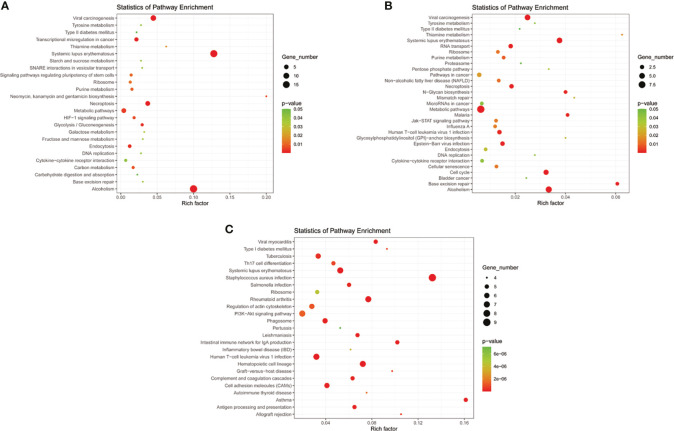
KEGG pathway analyses of important lncRNAs (intersection of all genes in selected module and differentially expressed lncRNAs) in **(A)** T2DM versus normal controls, **(B)** T2DM versus prediabetes and **(C)** prediabetes versus normal controls.

Eight models (black, blue, brown, green, grey, red, turquoise, yellow) were obtained through constructing co-expression networks (**Figure 1A**). The number of genes in different modules was shown in **Table 3**. Specially, the genes classified in grey model indicated that they were not assigned to any module. The correlation between genes was displayed in **Figures 2A, B** indicated the correlation between different modules. Meanwhile, by combining clinical data (gender, age, blood glucose and BMI) of T2DM patients and normal controls, we could get the relevant modules and corresponding genes. Based on the aims of the study, modules that are highly related to blood glucose have received attention. As shown in **Figure 3A**, the green module (*r*=0.7, *P*=0.01) was significantly related to blood glucose. Additionally, the results of differentially expressed lncRNAs between T2DM patients and normal controls were shown in **Figures 4A, B**, which revealing that 528 lncRNAs were up-regulated and 622 lncRNAs were down-regulated. Subsequently, we take the intersection of all genes in green module and differentially expressed lncRNAs to obtain key lncRNAs (**Table S1**). Then, the pathway analysis to the target genes corresponding to these lncRNAs was performed. **Figure 5A** indicated that Glycolysis/Gluconeogenesis, Metabolic pathways, Type II diabetes mellitus and other signaling pathway were enriched. We found hexokinase-3(HK3) was involved in all above pathways. The corresponding lncRNA of HK3 was ENST00000503273. All enriched signaling pathways of HK3 were displayed in **Tables S2** and **S5**.

**Table 3:** Number of lncRNAs contained in different modules.

Type 2 diabetes mellitus versus normal controls							
Module	black		blue		brown		green
Number	67		549		364		97
Module	grey		red		turquoise		yellow
Number	491		76		3230		126
***Type 2 diabetes mellitus versus prediabetes***							
Module	black	blue	brown	green	grey	magenta	
Number	44	1237	464	86	25	40	
Module	pink	purple	red	turquoise	yellow		
Number	44	34	76	2811	139		
***Prediabetes versus normal controls***							
Module	black		blue		brown		green
Number	46		1207		587		124
Module	grey		red		turquoise		yellow
Number	62		56		2592		326

### Type 2 Diabetes *Versus* Prediabetes

By constructing co-expression networks, eleven models (black, blue, brown, green, grey, magenta, pink, purple, red, turquoise, yellow) were obtained (**Figure 1B**). The number of genes in different modules was shown in **Table 3**. The correlation between genes was shown in **Figures 2C, D** indicated the correlation between different modules. Meanwhile, we could get the blood glucose related modules and corresponding genes *via* combining clinical data of T2DM and prediabetes patients. As shown in **Figure 3B**, the yellow module (*r*=0.69, *P*=0.01) was significantly related to blood glucose. Additionally, the results of differentially expressed lncRNAs between T2DM and prediabetes patients were displayed in **Figures 4C, D**, which containing 807 up-regulated lncRNAs and 809 down-regulated lncRNAs. Similarly, we take the intersection of all genes in yellow module and differentially expressed lncRNAs to obtain key lncRNAs (**Table S1**), and the pathway analysis was performed. **Figure 5B** indicated that Type II diabetes mellitus, Insulin resistance, Fc gamma R-mediated phagocytosis, Inflammatory mediator regulation of TRP channels and other signaling pathway were enriched. Protein kinase C-epsilon (PRKCE) was involved in all above pathways and corresponding lncRNA was ENST00000462720 and ENST00000480633. All enriched signaling pathways of PRKCE were shown in **Tables S3** and **S5**.

### Prediabetes *Versus* Normal Controls

Eight models were obtained by using WGCNA (**Figure 1C**). The number of genes in each module was shown in **Table 3**. The correlation between genes was shown in **Figures 2E, F** shown the correlation between different modules. Similarly, as shown in **Figure 3C**, the yellow module (*r*=-0.61, *P*=0.03) was significantly related to blood glucose. And the results of differentially expressed lncRNAs between prediabetes and Normal Controls were shown in **Figures 4E, F**. Subsequently, we used the same method to obtain key lncRNAs and conducted the pathway analysis (**Table S1**). **Figure 5C** indicated that PI3K-Akt signaling pathway, Cortisol synthesis and secretion, TNF signaling pathway and other signaling pathway were enriched. Activating transcription factor 6-β (ATF6B) was enriched in all above pathways and the corresponding lncRNA was ENST00000485392. All enriched signaling pathways of ATF6B were displayed in **Tables S4** and **S5**.

### Validation *via* qRT‐PCR, GEO Data Set and ROC Curve

**Figure 6:**
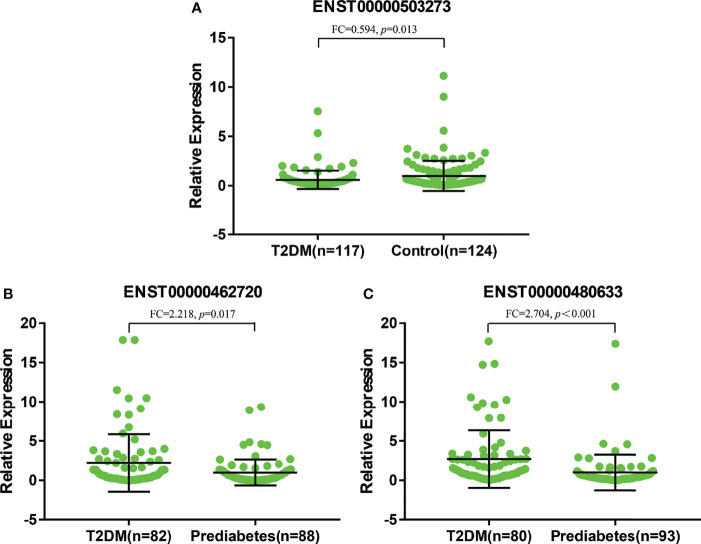
**(A)** represented relative expression of lncRNAs for ENST00000503273 between T2DM and normal controls. **(B, C)** represented relative expression of lncRNAs for ENST00000462720 and ENST00000480633 between T2DM and prediabetes, respectively.

**Figure 7:**
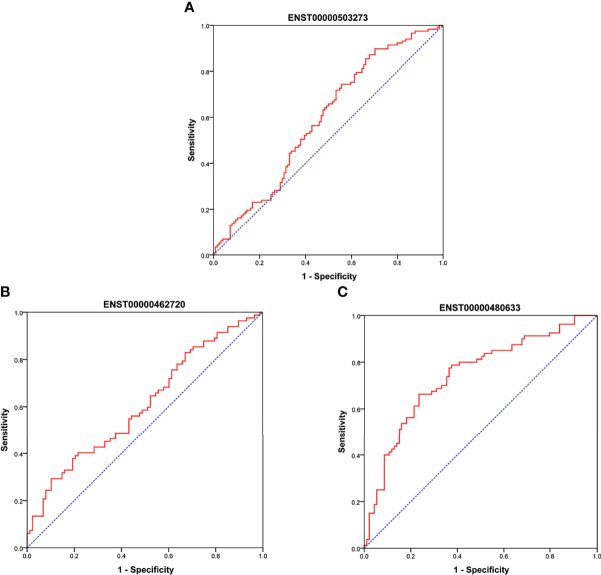
**(A)** showed ROC curve for ENST00000503273 between T2DM and normal controls. **(B, C)** showed ROC curve for ENST00000462720 and ENST00000480633 between T2DM and prediabetes, respectively.

As shown in **Figure 6A**, there were significant differences for ENST00000503273 between the T2DM patients and normal controls *via* the validation of qRT‐PCR (*z=-2.472, P=0.013*). Meanwhile, significant differences for ENST00000462720(*z=-2.389, P=0.017*) and ENST00000480633(*z=-5.477, P<0.001*) were observed between the T2DM and prediabetes patients based on **Figures 6B, C**, respectively. However, no significant difference was found for ENST00000485392 between the prediabetes and normal controls based on qRT‐PCR. Meanwhile, the corresponding genes of above lncRNAs were verified *via* GSE 130991 data set. 74 T2DM, 23 prediabetes patients and 112 controls were selected from the data set. However, the corresponding gene of ENST00000485392 was not detected in this array. The p-value and log2 fold change of all genes were shown in **Table S6**. Moreover, the ROC curve was used to evaluate the diagnostic power of above lncRNAs. Detailed situation was shown in **Table 4** and **Figure 7**.

**Table 4:** The Diagnostic Values of LncRNAs (ROC curve).

	Sensitivity (%)	Specificity (%)	AUC	*P*	*95%CI*
ENST00000503273	89.7	29.8	0.592	0.013	0.520-0.664
ENST00000462720	29.3	89.8	0.606	0.017	0.521-0.691
ENST00000480633	66.3	76.3	0.742	<0.001	0.667-0.816

## Discussion

The prevalence of diabetes in adults aged 18–99 years was estimated to be 8.4% in 2017 and predicted to rise to 9.9% in 2045. Meanwhile, there were 374 million people, equaling 7.7% of the world population, who have impaired glucose tolerance. Based on this, the healthcare expenditure due to hyperglycemia has brought a large social, financial and health system burden to the world (4). We used WGCNA to find the key lncRNA was ENST00000503273 and the corresponding mRNA was HK3 between T2DM patients and normal controls. Similarly, the key lncRNAs were ENST00000462720 and ENST00000480633, and the corresponding mRNA was PRKCE between T2DM and prediabetes patients. Moreover, the key lncRNA was ENST00000485392 and the corresponding mRNA was ATF6B between prediabetes and normal controls.

Hexokinase was involved in phosphorylation of glucose to produce glucose-6-phosphate, the initial step in glycolysis and most glucose metabolism pathways (24). When hungry, the body mobilizes stored fat to decompose and oxidize to produce large amounts of acetyl-CoA, which can be condensed with oxaloacetic acid to form citric acid to reduce glycolysis. Meanwhile, under fed conditions, glycolytic substrates appear to contribute at most 50% of the acetyl-CoA requirements for oxidation to fat (25). Previous studies have indicated that PRKCE could regulate Protein kinase C-delta (PRKCD), and PRKCD is related to the accumulation of triglycerides and the production of lipogenic enzymes in liver for mouse (26). Meanwhile, existing researches have shown that along with increased triglyceride and FFA levels, ATF6 protein was elevated after infusion (27). Therefore, these genes seem to be related to triglycerides or lipids.

Glycolysis pathway is one of the most critical pathways of glucose metabolism, and it is also a pathway that links the metabolism of glucose, fat and amino acids. For diabetes, the activities of hexokinase and glycogen synthase in the liver and skeletal muscle are reduced, resulting in hepatic glycogen increased and glycogen synthesis decreased, thus promoting blood glucose. Meanwhile, for patients with T2DM, insulin is relatively insufficient due to decreased insulin sensitivity in the liver, leading to the synthesis of glycolysis-related enzyme reduced and glycolysis weakened. Subsequently, the ability to metabolize glucose is lessened. Finally, it has a certain impact on the blood glucose balance (28, 29). Notably, there are a number of genes are related to insulin sensitivity. For example, an increase in HK3 could contribute to the improvement of insulin sensitivity (30). Meanwhile, previous studies have indicated that some synthase appears to stimulate HK3 expression to improve insulin sensitivity (31). Therefore, through the glycolysis pathway, the mechanism of HK3 affecting the development of T2DM could be improving insulin sensitivity.

Early study found that overexpression of PRKCE could be associated with the development of insulin resistance by decreasing the insulin receptors in animals (32). The current literature indicated that increased PRKCE activation was related to marked increases TG in liver, which further led to insulin resistance (33). Meanwhile, PRKCE could also impairs insulin signaling and its ability to activate glycogen synthesis and inhibit neoglucogenesis, resulting in insulin resistance (34). It is well known that insulin resistance is a core defect in T2DM (35). Besides, PRKCE is a critical gene in steatosis for NAFLD patients, and NAFLD is a risk factor for T2DM (36, 37). Moreover, the association of PRKCE with insulin granules is essential for insulin secretion (38). Therefore, it seems that PRKCE can cause the further development of T2DM through a variety of ways. Meanwhile, the activation of protein kinase C (PKC) is considered to be one of the ways that hyperglycemia leads to the development of diabetic vascular complications and other diabetic complications (39, 40).

PI3K-Akt signaling pathway is related to endoplasmic reticulum (ER) stress (41). ER stress is a key phenomenon in the obesity- and T2DM-associated adverse metabolic outcomes, including insulin resistance in key metabolic organs (42). Studies have indicated that ATF6 is a key transcription factor regulating ER stress (43). Mammals express two homologous ATF6(ATF6α and ATF6β), and ATF6β contributes to adipogenic processes (44). Correspondingly, PI3K-AKT signaling pathway promotes lipid biosynthesis and inhibits lipolysis (45). Therefore, ATF6β could promote the production of adipocytokines, and then the adipocytokines lead to insulin resistance *via* blocking PI3K-AKT-mediated inhibition of lipolysis attenuating the capacity of glucose utilization (45). Meanwhile, ATF6 could increase expression of TNF-α and other inflammatory cytokines in response to ER stress (46). The inflammatory cytokines enhance lipolysis by reducing perilipin and fat-specific protein 27 levels, and then the hepatic insulin-AKT signaling was impaired (47–49). Therefore, ATF6β could involve in insulin resistance through PI3K-Akt signaling pathway, which also included the role of TNF-α. and in turn, insulin resistance aggravates the PI3K-AKT pathway, forming a vicious circle (45). Specially, literatures have indicated that the genetic variation in ATF6 is related to prediabetes in the Chinese Han population (50). However, no significant difference was found for its corresponding lncRNA based on qRT‐PCR in our study, more sample was needed in the future.

This research has some limitations. In order to get reliable results, a larger sample size will be needed. Moreover, although we have used qRT-PCR to verify the key lncRNA, more molecular biology experiments and functional studies are required to help validate our findings in the future.

## Conclusion

For hypertriglyceridemia patients with different blood glucose levels, ENST00000503273, ENST00000462720 and ENST00000480633 could be potential biomarkers of T2DM.

## Data Availability Statement

The original contributions presented in the study are publicly available. This data can be found here: NCBI, GEO, GSE193436.

## Ethics Statement

The studies involving human participants were reviewed and approved by the Ethics Committee of the Public Health of the Jilin University. The patients/participants provided their written informed consent to participate in this study.

## Author Contributions

BL, WC, and SY made the study design. SY, MS, LG, and NY conducted the study. SY, MS, TF, and YY analyzed the data and wrote the manuscript. SY, MS, XL, and WH participated amending the manuscript. All authors contributed to the article and approved the submitted version.

## Funding

This work was supported by the National Natural Science Foundation of China (81973129) and the Graduate Innovative Research Program of Jilin University (101832020CX265).

## Conflict of Interest

The authors declare that the research was conducted in the absence of any commercial or financial relationships that could be construed as a potential conflict of interest.

## Publisher’s Note

All claims expressed in this article are solely those of the authors and do not necessarily represent those of their affiliated organizations, or those of the publisher, the editors and the reviewers. Any product that may be evaluated in this article, or claim that may be made by its manufacturer, is not guaranteed or endorsed by the publisher.
