# Supplementary material for: Identification of Key LncRNAs and Pathways in Prediabetes and Type 2 Diabetes Mellitus for Hypertriglyceridemia Patients Based on Weighted Gene Co-Expression Network Analysis
Source: Front Endocrinol (Lausanne). 2022 Jan 24;12:800123. doi: 10.3389/fendo.2021.800123 (PMC8818867; doi:10.3389/fendo.2021.800123)
Supplement: Supplementary file 5 [file Table_1.docx]

Table S1 Differentially Expressed Transcripts (DETs) of Each Module Related to Blood Glucose

| Module | DETs |
| --- | --- |
| Green Module  (Type 2 Diabetes versus Normal Controls*)* | ENST00000505281 |
|  | ENST00000513640 |
|  | ENST00000510804 |
|  | ENST00000476585 |
|  | ENST00000492182 |
|  | ENST00000494468 |
|  | ENST00000460037 |
|  | ENST00000469337 |
|  | ENST00000475744 |
|  | ENST00000481214 |
|  | ENST00000481073 |
|  | ENST00000495003 |
|  | ENST00000468259 |
|  | ENST00000489954 |
|  | ENST00000461407 |
|  | ENST00000561017 |
|  | ENST00000557983 |
|  | ENST00000557793 |
|  | ENST00000510376 |
|  | ENST00000503273 |
|  | ENST00000505229 |
|  | ENST00000515488 |
|  | ENST00000505879 |
|  | ENST00000417486 |
|  | ENST00000554671 |
|  | ENST00000554377 |
|  | ENST00000556937 |
|  | ENST00000553850 |
|  | ENST00000554851 |
|  | ENST00000556584 |
|  | ENST00000555724 |
|  | ENST00000483402 |
|  | ENST00000463018 |
|  | TCONS_00007033 |
|  | ENST00000472787 |
|  | ENST00000498720 |
|  | ENST00000482601 |
|  | ENST00000484303 |
|  | ENST00000482961 |
|  | ENST00000491448 |
|  | ENST00000476958 |
|  | ENST00000467352 |
|  | TCONS_00235287 |
|  | ENST00000460481 |
|  | ENST00000494601 |
|  | ENST00000392584 |
|  | ENST00000468069 |
|  | ENST00000490299 |
|  | ENST00000491616 |
|  | ENST00000521631 |
|  | ENST00000522808 |
|  | ENST00000520073 |
|  | ENST00000523994 |
|  | TCONS_00039273 |
|  | TCONS_00047799 |
|  | TCONS_00047798 |
|  | TCONS_00047797 |
|  | TCONS_00047796 |
|  | TCONS_00039274 |
|  | ENST00000606752 |
|  | ENST00000443016 |
|  | ENST00000488893 |
|  | ENST00000419600 |
|  | ENST00000494037 |
|  | ENST00000541592 |
|  | ENST00000537096 |
|  | ENST00000540565 |
|  | ENST00000537853 |
|  | ENST00000530422 |
|  | TCONS_00334653 |
|  | ENST00000541782 |
|  | TCONS_00117764 |
|  | TCONS_00117765 |
|  | TCONS_00117762 |
|  | TCONS_00117763 |
|  | TCONS_00117761 |
| Yellow Module  (Type 2 Diabetes versus Prediabetes) | ENST00000496205 |
|  | ENST00000482590 |
|  | ENST00000461326 |
|  | ENST00000492793 |
|  | ENST00000472679 |
|  | ENST00000473565 |
|  | ENST00000481641 |
|  |  |
|  | ENST00000464454 |
|  | ENST00000461981 |
|  | ENST00000475073 |
|  | ENST00000461805 |
|  | ENST00000493649 |
|  | ENST00000490133 |
|  | ENST00000474067 |
|  | ENST00000498463 |
|  | ENST00000496855 |
|  | ENST00000492626 |
|  | ENST00000494402 |
|  | ENST00000471744 |
|  | TCONS_00354277 |
|  | ENST00000482558 |
|  | ENST00000475800 |
|  | ENST00000476585 |
|  | ENST00000492182 |
|  | ENST00000494468 |
|  | ENST00000460037 |
|  | ENST00000469337 |
|  | ENST00000475744 |
|  | ENST00000481214 |
|  | ENST00000481073 |
|  | ENST00000495003 |
|  | ENST00000468259 |
|  | ENST00000489954 |
|  | ENST00000461407 |
|  | ENST00000561017 |
|  | ENST00000557983 |
|  | ENST00000557793 |
|  | ENST00000553278 |
|  | ENST00000555550 |
|  | ENST00000556256 |
|  | ENST00000556486 |
|  | ENST00000555953 |
|  | ENST00000556534 |
|  | ENST00000554819 |
|  | ENST00000553280 |
|  | ENST00000554486 |
|  | ENST00000556413 |
|  | ENST00000554671 |
|  | ENST00000554377 |
|  | ENST00000556937 |
|  | ENST00000553850 |
|  | ENST00000554851 |
|  | ENST00000556584 |
|  | ENST00000555724 |
|  | ENST00000563893 |
|  | ENST00000567459 |
|  | ENST00000562041 |
|  | ENST00000478904 |
|  | ENST00000488761 |
|  | ENST00000496670 |
|  | ENST00000483608 |
|  | TCONS_00235406 |
|  | ENST00000461711 |
|  | ENST00000481488 |
|  | ENST00000490200 |
|  | ENST00000495601 |
|  | ENST00000483402 |
|  | ENST00000463018 |
|  | TCONS_00007033 |
|  | ENST00000472787 |
|  | ENST00000498720 |
|  | ENST00000482601 |
|  | ENST00000484303 |
|  | ENST00000482961 |
|  | ENST00000491448 |
|  | ENST00000476958 |
|  | ENST00000467352 |
|  | TCONS_00235287 |
|  | ENST00000496860 |
|  | ENST00000478672 |
|  | ENST00000474507 |
|  | ENST00000461592 |
|  | ENST00000483394 |
|  | TCONS_00334574 |
|  | ENST00000460481 |
|  | ENST00000494601 |
|  | ENST00000392584 |
|  | ENST00000497500 |
|  | ENST00000486136 |
|  | ENST00000487595 |
|  | ENST00000485250 |
|  | ENST00000477844 |
|  |  |
|  | ENST00000489145 |
|  | ENST00000490590 |
|  | ENST00000495505 |
|  | ENST00000487293 |
|  | ENST00000488951 |
|  | ENST00000488516 |
|  | ENST00000480491 |
|  | ENST00000484879 |
|  | ENST00000525036 |
|  | ENST00000531171 |
|  | ENST00000521631 |
|  | ENST00000522808 |
|  | ENST00000520073 |
|  | ENST00000523994 |
|  | TCONS_00039273 |
|  | TCONS_00047799 |
|  | TCONS_00047798 |
|  | TCONS_00047797 |
|  | TCONS_00047796 |
|  | TCONS_00039274 |
|  | ENST00000606752 |
|  | ENST00000443016 |
|  | ENST00000497330 |
|  | ENST00000472844 |
|  | ENST00000479162 |
|  | ENST00000461862 |
|  | ENST00000459799 |
|  | ENST00000471039 |
|  | ENST00000497147 |
|  | ENST00000462235 |
|  | ENST00000463044 |
|  | ENST00000436236 |
|  | ENST00000423527 |
|  | ENST00000488151 |
|  | ENST00000453168 |
|  | TCONS_00282826 |
|  | TCONS_00346834 |
|  | TCONS_00346835 |
|  | TCONS_00346832 |
|  | ENST00000514603 |
|  | TCONS_00301284 |
|  | ENST00000509346 |
|  | ENST00000644398 |
|  | ENST00000510771 |
|  | ENST00000643254 |
|  | TCONS_00309841 |
|  | TCONS_00309820 |
|  | TCONS_00309843 |
|  | ENST00000533665 |
|  | ENST00000528580 |
|  | ENST00000528963 |
|  | ENST00000532660 |
|  | TCONS_00390812 |
|  | ENST00000526007 |
|  | ENST00000534737 |
|  | ENST00000526985 |
|  | ENST00000530422 |
|  | ENST00000554988 |
|  | TCONS_00117764 |
|  | TCONS_00117765 |
|  | TCONS_00117762 |
|  | TCONS_00117763 |
|  | TCONS_00117761 |
| Yellow Module  (Prediabetes versus Normal Controls) | ENST00000490329 |
|  | ENST00000374089 |
|  | ENST00000447808 |
|  | ENST00000563893 |
|  | ENST00000567459 |
|  | ENST00000562041 |
|  | ENST00000461176 |
|  | ENST00000473963 |
|  | ENST00000465294 |
|  | ENST00000473571 |
|  | ENST00000473374 |
|  | ENST00000476304 |
|  | ENST00000474947 |
|  | ENST00000462090 |
|  | ENST00000475011 |
|  | ENST00000473620 |
|  | ENST00000464438 |
|  | ENST00000487431 |
|  | ENST00000495728 |
|  | ENST00000485459 |
|  | ENST00000491266 |
|  | ENST00000496055 |
|  | ENST00000497648 |
|  | ENST00000470201 |
|  | ENST00000467748 |
|  | ENST00000471759 |
|  | ENST00000461653 |
|  | ENST00000489450 |
|  | ENST00000462042 |
|  | ENST00000473406 |
|  | ENST00000459697 |
|  | ENST00000467168 |
|  | ENST00000491035 |
|  | ENST00000485196 |
|  | ENST00000479366 |
|  | TCONS_00416764 |
|  | ENST00000492572 |
|  | ENST00000482732 |
|  | ENST00000489200 |
|  | TCONS_00039273 |
|  | TCONS_00047799 |
|  | TCONS_00047798 |
|  | TCONS_00047797 |
|  | TCONS_00047796 |
|  | TCONS_00039274 |
|  | ENST00000606752 |
|  | ENST00000443016 |
|  | ENST00000585176 |
|  | ENST00000578115 |
|  | ENST00000578069 |
|  | ENST00000583515 |
|  | ENST00000493861 |
|  | ENST00000484870 |
|  | ENST00000492156 |
|  | ENST00000652375 |
|  | ENST00000489047 |
|  | ENST00000651127 |
|  | ENST00000651597 |
|  | ENST00000477640 |
|  | ENST00000469164 |
|  | ENST00000509168 |
|  | TCONS_00299353 |
|  | TCONS_00307682 |
|  | TCONS_00307683 |
|  | TCONS_00307680 |
|  | TCONS_00307681 |
|  | TCONS_00307684 |
|  | ENST00000507167 |
|  | ENST00000511753 |
|  | ENST00000572899 |
|  | ENST00000574970 |
|  | ENST00000576925 |
|  | ENST00000590218 |
|  | ENST00000571735 |
|  | ENST00000460633 |
|  | ENST00000468844 |
|  | ENST00000477403 |
|  | TCONS_00227327 |
|  | ENST00000495018 |
|  | ENST00000469562 |
|  | ENST00000443631 |
|  | ENST00000473957 |
|  | ENST00000485263 |
|  | ENST00000478388 |
|  | ENST00000485543 |
|  | ENST00000486992 |
|  | ENST00000478438 |
|  | ENST00000468936 |
|  | ENST00000648821 |
|  | ENST00000490071 |
|  | ENST00000461632 |
|  | ENST00000496065 |
|  | ENST00000468237 |
|  | ENST00000496560 |
|  | ENST00000463249 |
|  | ENST00000474912 |
|  | ENST00000472218 |
|  | ENST00000479802 |
|  | ENST00000496813 |
|  | ENST00000474626 |
|  | ENST00000490191 |
|  | ENST00000476222 |
|  | ENST00000616815 |
|  | ENST00000653733 |
|  | TCONS_00040285 |
|  | TCONS_00040286 |
|  | TCONS_00398820 |
