# Supplementary material for: Identification of Key LncRNAs and Pathways in Prediabetes and Type 2 Diabetes Mellitus for Hypertriglyceridemia Patients Based on Weighted Gene Co-Expression Network Analysis
Source: Front Endocrinol (Lausanne). 2022 Jan 24;12:800123. doi: 10.3389/fendo.2021.800123 (PMC8818867; doi:10.3389/fendo.2021.800123)
Supplement: Supplementary file 6 [file Table_2.docx]

Table S2 The results of KEGG in Type 2 Diabetes versus Normal Controls

| Pathway | *P* | Input |
| --- | --- | --- |
| Alcoholism | 1.09E-30 | HIST1H4L\|HIST1H4J\|HIST1H4K\|HAT1\|HIST1H2BM\|H2AFJ\|HIST1H2AJ\|HIST1H2AL\|HIST1H2AK\|HIST1H2BO\|HIST1H2AM\|HIST4H4\|HIST1H2AI\|HIST1H2BN\|HIST1H2BL\|HIST1H3I\|HIST1H3J\|HIST1H3H |
| Systemic lupus erythematosus | 1.18E-30 | HIST1H4L\|HIST1H4J\|HIST1H4K\|H2AFJ\|HIST1H2BM\|HIST1H2AJ\|HIST1H2AL\|HIST1H2AK\|HIST1H2BO\|HIST1H2AM\|HIST4H4\|HIST1H2AI\|HIST1H2BN\|HIST1H2BL\|HIST1H3I\|HIST1H3J\|HIST1H3H |
| Viral carcinogenesis | 1.44E-12 | HIST1H4J\|HIST1H4K\|HIST1H4L\|HIST1H2BO\|HIST1H2BM\|HIST4H4\|HIST1H2BN\|HIST1H2BL\|CHD4 |
| Necroptosis | 2.99E-08 | HIST1H2AL\|HIST1H2AK\|HIST1H2AJ\|HIST1H2AI\|HIST1H2AM\|H2AFJ |
| Transcriptional misregulation in cancer | 5.64E-05 | TSPAN7\|HIST1H3I\|HIST1H3J\|HIST1H3H |
| Endocytosis | 0.002516466 | RAB11FIP1\|DNAJC6\|FGFR4 |
| Glycolysis / Gluconeogenesis | 0.002695508 | GAPDH\|HK3 |
| Metabolic pathways | 0.004567009 | GAPDH\|AK4\|MGAT2\|HK3\|GUCY2C\|FAH |
| Neomycin, kanamycin and gentamicin biosynthesis | 0.00654953 | HK3 |
| HIF-1 signaling pathway | 0.006623553 | GAPDH\|HK3 |
| Carbon metabolism | 0.007575388 | GAPDH\|HK3 |
| Purine metabolism | 0.009245385 | AK4\|GUCY2C |
| Signaling pathways regulating pluripotency of stem cells | 0.010631453 | WNT8A\|FGFR4 |
| Ribosome | 0.012561683 | RPS29\|RPL36AL |
| Thiamine metabolism | 0.018448355 | AK4 |
| Galactose metabolism | 0.034449941 | HK3 |
| Base excision repair | 0.03656414 | POLE2 |
| Fructose and mannose metabolism | 0.03656414 | HK3 |
| SNARE interactions in vesicular transport | 0.037619543 | VAMP1 |
| DNA replication | 0.039726963 | POLE2 |
| Tyrosine metabolism | 0.039726963 | FAH |
| Starch and sucrose metabolism | 0.039726963 | HK3 |
| Cytokine-cytokine receptor interaction | 0.041721068 | LEPR\|CD27 |
| Carbohydrate digestion and absorption | 0.048111658 | HK3 |
| Type II diabetes mellitus | 0.050196633 | HK3 |
| Human papillomavirus infection | 0.051234959 | WNT8A\|CHD4 |
| Nucleotide excision repair | 0.051237447 | POLE2 |
| Amino sugar and nucleotide sugar metabolism | 0.052277147 | HK3 |
| N-Glycan biosynthesis | 0.05435321 | MGAT2 |
| Basal cell carcinoma | 0.067739751 | WNT8A |
| Central carbon metabolism in cancer | 0.073855554 | HK3 |
| Adipocytokine signaling pathway | 0.073855554 | LEPR |
| Biosynthesis of amino acids | 0.079932163 | GAPDH |
| Melanogenesis | 0.105817183 | WNT8A |
| Ribosome biogenesis in eukaryotes | 0.109735834 | XPO1 |
| Pathways in cancer | 0.11508655 | WNT8A\|FGFR4 |
| Thyroid hormone signaling pathway | 0.123319466 | MED17 |
| AMPK signaling pathway | 0.124281941 | LEPR |
| Spliceosome | 0.138595817 | WBP11 |
| Insulin signaling pathway | 0.140486982 | HK3 |
| Breast cancer | 0.149882146 | WNT8A |
| Non-alcoholic fatty liver disease (NAFLD) | 0.151749105 | LEPR |
| Gastric cancer | 0.151749105 | WNT8A |
| mTOR signaling pathway | 0.155471016 | WNT8A |
| Hippo signaling pathway | 0.156398999 | WNT8A |
| Cushing syndrome | 0.157325985 | WNT8A |
| Wnt signaling pathway | 0.161946015 | WNT8A |
| Jak-STAT signaling pathway | 0.163787092 | LEPR |
| RNA transport | 0.166541302 | XPO1 |
| Influenza A | 0.168372517 | XPO1 |
| Hepatocellular carcinoma | 0.16928665 | WNT8A |
| Alzheimer disease | 0.172023165 | GAPDH |
| Axon guidance | 0.181081442 | UNC5A |
| Proteoglycans in cancer | 0.200670267 | WNT8A |
| Rap1 signaling pathway | 0.206806539 | FGFR4 |
| Regulation of actin cytoskeleton | 0.210292297 | FGFR4 |
| Human T-cell leukemia virus 1 infection | 0.214628458 | XPO1 |
| Ras signaling pathway | 0.225793933 | FGFR4 |
| MAPK signaling pathway | 0.277744333 | FGFR4 |
| MicroRNAs in cancer | 0.280925256 | ZEB2 |
| Neuroactive ligand-receptor interaction | 0.311230804 | LEPR |
| PI3K-Akt signaling pathway | 0.323299699 | FGFR4 |
| Olfactory transduction | 0.390142867 | OR2B2 |
