# Supplementary material for: Identification of Key LncRNAs and Pathways in Prediabetes and Type 2 Diabetes Mellitus for Hypertriglyceridemia Patients Based on Weighted Gene Co-Expression Network Analysis
Source: Front Endocrinol (Lausanne). 2022 Jan 24;12:800123. doi: 10.3389/fendo.2021.800123 (PMC8818867; doi:10.3389/fendo.2021.800123)
Supplement: Supplementary file 7 [file Table_3.docx]

Table S3 The results of KEGG in Type 2 Diabetes versus Prediabetes

| Pathway | *P* | Input |
| --- | --- | --- |
| Alcoholism | 5.47E-08 | HIST1H2AH\|HIST1H2BK\|CCND39\|  HIST1H4I\|HIST1H2BJ\|HAT1 |
| Systemic lupus erythematosus | 4.30E-07 | HIST1H2BJ\|CCND39\|HIST1H2AH\|  HIST1H4I\|HIST1H2BK |
| Viral carcinogenesis | 3.09E-06 | HIST1H2BJ\|RB1\|HIST1H4I\|CCND3\|HIST1H2BK |
| Cell cycle | 1.20E-05 | RB1\|ORC4\|CCND3\|ORC2 |
| Metabolic pathways | 2.15E-05 | AK4\|PRPS1L1\|MGAT2\|OPLAH\|CYC1\|  STT3B\|FAH\|CDIPT\|GPAA1 |
| Base excision repair | 0.00068 | POLE2\|PARP2 |
| Necroptosis | 0.000793 | CCND39\|HIST1H2AH\|PARP2 |
| RNA transport | 0.000836 | XPO1\|NUPL2\|NUP35 |
| Malaria | 0.001442 | GYPA\|GYPB |
| Epstein-Barr virus infection | 0.001461 | RB1\|CCND3\|PSMD4 |
| N-Glycan biosynthesis | 0.001499 | MGAT2\|STT3B |
| Human T-cell leukemia virus 1 infection | 0.001859 | RB1\|XPO1\|CCND3 |
| Purine metabolism | 0.009245 | PRPS1L1\|AK4 |
| Non-alcoholic fatty liver disease (NAFLD) | 0.011953 | CYC1\|LEPR |
| Ribosome | 0.012562 | RPS29\|RPL36AL |
| Cellular senescence | 0.01366 | RB1\|CCND3 |
| Jak-STAT signaling pathway | 0.013981 | LEPR\|CCND3 |
| Influenza A | 0.014798 | XPO1\|CCND3 |
| Thiamine metabolism | 0.018448 | AK4 |
| Pathways in cancer | 0.020514 | RB1\|CCND3\|LPAR6 |
| Mismatch repair | 0.025948 | PMS2 |
| Glycosylphosphatidylinositol (GPI)-anchor biosynthesis | 0.02808 | GPAA1 |
| Endocytosis | 0.029797 | RAB11FIP1\|DNAJC6 |
| Pentose phosphate pathway | 0.033391 | PRPS1L1 |
| Tyrosine metabolism | 0.039727 | FAH |
| DNA replication | 0.039727 | POLE2 |
| Cytokine-cytokine receptor interaction | 0.041721 | LEPR\|ACVR2A |
| MicroRNAs in cancer | 0.042998 | ZEB2\|PRKCE |
| Bladder cancer | 0.044976 | RB1 |
| Proteasome | 0.049155 | PSMD4 |
| Type II diabetes mellitus | 0.050197 | PRKCE |
| Human papillomavirus infection | 0.051235 | RB1\|CCND3 |
| Nucleotide excision repair | 0.051237 | POLE2 |
| Neuroactive ligand-receptor interaction | 0.053446 | LEPR\|LPAR6 |
| PI3K-Akt signaling pathway | 0.05797 | CCND3\|LPAR6 |
| Fanconi anemia pathway | 0.058492 | PMS2 |
| Legionellosis | 0.059524 | CLK1 |
| Glutathione metabolism | 0.060555 | OPLAH |
| Non-small cell lung cancer | 0.070803 | RB1 |
| Adipocytokine signaling pathway | 0.073856 | LEPR |
| Melanoma | 0.076899 | RB1 |
| p53 signaling pathway | 0.076899 | CCND3 |
| Inositol phosphate metabolism | 0.078922 | CDIPT |
| Pancreatic cancer | 0.079932 | RB1 |
| Glioma | 0.079932 | RB1 |
| Biosynthesis of amino acids | 0.079932 | PRPS1L1 |
| Chronic myeloid leukemia | 0.080941 | RB1 |
| RNA degradation | 0.083962 | EXOSC4 |
| Cardiac muscle contraction | 0.090972 | CYC1 |
| Small cell lung cancer | 0.097929 | RB1 |
| TGF-beta signaling pathway | 0.098919 | ACVR2A |
| Fc gamma R-mediated phagocytosis | 0.098919 | PRKCE |
| Prostate cancer | 0.101882 | RB1 |
| Glycerophospholipid metabolism | 0.101882 | CDIPT |
| Hematopoietic cell lineage | 0.101882 | GYPA |
| Aldosterone synthesis and secretion | 0.102867 | PRKCE |
| Endocrine resistance | 0.102867 | RB1 |
| Phosphatidylinositol signaling system | 0.103852 | CDIPT |
| Inflammatory mediator regulation of TRP channels | 0.104835 | PRKCE |
| AGE-RAGE signaling pathway in diabetic complications | 0.104835 | PRKCE |
| Ribosome biogenesis in eukaryotes | 0.109736 | XPO1 |
| Insulin resistance | 0.112664 | PRKCE |
| Carbon metabolism | 0.121391 | PRPS1L1 |
| Thyroid hormone signaling pathway | 0.123319 | MED17 |
| Sphingolipid signaling pathway | 0.123319 | PRKCE |
| AMPK signaling pathway | 0.124282 | LEPR |
| Vascular smooth muscle contraction | 0.135751 | PRKCE |
| Oxidative phosphorylation | 0.136701 | CYC1 |
| Apoptosis | 0.139542 | PARP2 |
| Apelin signaling pathway | 0.140487 | PRKCE |
| Measles | 0.141431 | CCND3 |
| Fluid shear stress and atherosclerosis | 0.142374 | ACVR2A |
| Signaling pathways regulating pluripotency of stem cells | 0.143316 | ACVR2A |
| Parkinson disease | 0.145197 | CYC1 |
| Breast cancer | 0.149882 | RB1 |
| Phospholipase D signaling pathway | 0.150816 | LPAR6 |
| Gastric cancer | 0.151749 | RB1 |
| Hippo signaling pathway | 0.156399 | CCND3 |
| Cushing syndrome | 0.157326 | RB1 |
| Hepatitis C | 0.157326 | RB1 |
| Wnt signaling pathway | 0.161946 | CCND3 |
| Hepatitis B | 0.164706 | RB1 |
| Protein processing in endoplasmic reticulum | 0.166541 | STT3B |
| cGMP-PKG signaling pathway | 0.168373 | PRKCE |
| Hepatocellular carcinoma | 0.169287 | RB1 |
| Tight junction | 0.171112 | PRKCE |
| Alzheimer disease | 0.172023 | CYC1 |
| Axon guidance | 0.181081 | SEMA6C |
| Kaposi sarcoma-associated herpesvirus infection | 0.185574 | RB1 |
| Huntington disease | 0.191824 | CYC1 |
| Focal adhesion | 0.197143 | CCND3 |
| Regulation of actin cytoskeleton | 0.210292 | NCKAP1 |
| Human cytomegalovirus infection | 0.219801 | RB1 |
| Thermogenesis | 0.224941 | CYC1 |
| Olfactory transduction | 0.390143 | OR11H4 |
