# Supplementary material for: Identification of Key LncRNAs and Pathways in Prediabetes and Type 2 Diabetes Mellitus for Hypertriglyceridemia Patients Based on Weighted Gene Co-Expression Network Analysis
Source: Front Endocrinol (Lausanne). 2022 Jan 24;12:800123. doi: 10.3389/fendo.2021.800123 (PMC8818867; doi:10.3389/fendo.2021.800123)
Supplement: Supplementary file 8 [file Table_4.docx]

Table S4 The results of KEGG in Prediabetes versus Normal Controls

| Pathway | *P* | Input |
| --- | --- | --- |
| Staphylococcus aureus infection | 3.57E-15 | ITGB2\|HLA-DQB1\|HLA-DQA2\|C2\|HLA-DRB1\|CFB\|HLA-DQA1\|C4A\|C4B |
| Rheumatoid arthritis | 1.67E-10 | CSF2\|ITGB2\|HLA-DQB1\|HLA-DQA2\|HLA-DQA1\|HLA-DRB1\|ATP6AP1 |
| Hematopoietic cell lineage | 2.55E-10 | IL3\|CSF2\|HLA-DQB1\|HLA-DQA2\|HLA-DQA1\|HLA-DRB1\|EPO |
| Systemic lupus erythematosus | 2.06E-09 | HLA-DQB1\|HLA-DQA2\|C2\|HLA-DRB1\|HLA-DQA1\|C4A\|C4B |
| Asthma | 2.54E-09 | HLA-DQA1\|HLA-DRB1\|IL3\|HLA-DQB1\|HLA-DQA2 |
| Intestinal immune network for IgA production | 2.09E-08 | HLA-DQA1\|HLA-DRB1\|MADCAM1\|HLA-DQB1\|HLA-DQA2 |
| Viral myocarditis | 5.38E-08 | HLA-DQA1\|HLA-DRB1\|ITGB2\|HLA-DQB1\|HLA-DQA2 |
| Human T-cell leukemia virus 1 infection | 5.64E-08 | CSF2\|ITGB2\|HLA-DQB1\|HLA-DQA2\|HLA-DQA1\|HLA-DRB1\|ATF6B |
| Cell adhesion molecules (CAMs) | 1.28E-07 | ITGB2\|MADCAM1\|HLA-DQB1\|HLA-DQA2\|HLA-DQA1\|HLA-DRB1 |
| Leishmaniasis | 1.44E-07 | HLA-DQA1\|HLA-DRB1\|ITGB2\|HLA-DQB1\|HLA-DQA2 |
| Phagosome | 1.61E-07 | ITGB2\|HLA-DQB1\|HLA-DQA2\|HLA-DQA1\|HLA-DRB1\|ATP6AP1 |
| Antigen processing and presentation | 1.74E-07 | HLA-DQA1\|HLA-DRB1\|TAPBP\|HLA-DQB1\|HLA-DQA2 |
| Complement and coagulation cascades | 1.97E-07 | C2\|CFB\|ITGB2\|C4A\|C4B |
| Salmonella infection | 2.49E-07 | FLNA\|CSF2\|PFN4\|ARPC5L\|PKN3 |
| Tuberculosis | 4.06E-07 | ITGB2\|HLA-DQB1\|HLA-DQA2\|HLA-DQA1\|HLA-DRB1\|ATP6AP1 |
| Allograft rejection | 5.26E-07 | HLA-DQA1\|HLA-DRB1\|HLA-DQB1\|HLA-DQA2 |
| Graft-versus-host disease | 6.97E-07 | HLA-DQA1\|HLA-DRB1\|HLA-DQB1\|HLA-DQA2 |
| Th17 cell differentiation | 8.28E-07 | HLA-DQA1\|HLA-DRB1\|RXRB\|HLA-DQB1\|HLA-DQA2 |
| Type I diabetes mellitus | 8.33E-07 | HLA-DQA1\|HLA-DRB1\|HLA-DQB1\|HLA-DQA2 |
| Regulation of actin cytoskeleton | 1.12E-06 | ITGB2\|FGF22\|PFN4\|SLC9A1\|MYH10\|ARPC5L |
| PI3K-Akt signaling pathway | 1.32E-06 | IL3\|TNXB\|GNB2\|EPO\|FGF22\|PKN3\|ATF6B |
| Autoimmune thyroid disease | 1.82E-06 | HLA-DQA1\|HLA-DRB1\|HLA-DQB1\|HLA-DQA2 |
| Inflammatory bowel disease (IBD) | 3.94E-06 | HLA-DQA1\|HLA-DRB1\|HLA-DQB1\|HLA-DQA2 |
| Ribosome | 4.50E-06 | RPS18\|RPL35\|RPL26\|61ACADM\|RPL39 |
| Pertussis | 7.11E-06 | C2\|ITGB2\|C4A\|C4B |
| Metabolic pathways | 1.04E-05 | KYAT1\|CYP21A2\|NDUFA1\|ASS1\|ACADM\|ATP6AP1\|B3GALT4\|TKTL1\|CDIPT\|ACSL6\|HSD17B8 |
| Th1 and Th2 cell differentiation | 1.47E-05 | HLA-DQA1\|HLA-DRB1\|HLA-DQB1\|HLA-DQA2 |
| Epstein-Barr virus infection | 1.63E-05 | HLA-DQA1\|HLA-DRB1\|TAPBP\|HLA-DQB1\|HLA-DQA2 |
| Toxoplasmosis | 3.20E-05 | HLA-DQA1\|HLA-DRB1\|HLA-DQB1\|HLA-DQA2 |
| Apoptosis | 6.44E-05 | DAXX\|SPTAN1\|IL3\|ENDOG |
| Fatty acid metabolism | 0.000109 | ACSL6\|HSD17B8\|ACADM |
| Herpes simplex virus 1 infection | 0.000113 | HLA-DQB1\|HLA-DQA2\|DAXX\|HLA-DRB1\|HLA-DQA1\|TAPBP |
| Influenza A | 0.000139 | HLA-DQA1\|HLA-DRB1\|HLA-DQB1\|HLA-DQA2 |
| PPAR signaling pathway | 0.000246 | RXRB\|ACSL6\|ACADM |
| Fatty acid biosynthesis | 0.000428 | ACSL6\|HSD17B8 |
| Pathways in cancer | 0.001353 | FGF22\|IL3\|RXRB\|GNB2\|EPO |
| Jak-STAT signaling pathway | 0.002086 | IL3\|CSF2\|EPO |
| RNA transport | 0.002195 | UPF3B\|POP7\|EEF1A2 |
| Fatty acid degradation | 0.002272 | ACSL6\|ACADM |
| Transcriptional misregulation in cancer | 0.003062 | IL3\|CSF2\|RXRB |
| Legionellosis | 0.003465 | ITGB2\|EEF1A2 |
| Steroid hormone biosynthesis | 0.004086 | CYP21A2\|HSD17B8 |
| Rap1 signaling pathway | 0.004279 | FGF22\|ITGB2\|PFN4 |
| Cortisol synthesis and secretion | 0.004754 | CYP21A2\|ATF6B |
| Acute myeloid leukemia | 0.004893 | IL3\|CSF2 |
| Human cytomegalovirus infection | 0.005169 | TAPBP\|ATF6B\|GNB2 |
| Shigellosis | 0.005177 | PFN4\|ARPC5L |
| Fc epsilon RI signaling pathway | 0.005177 | IL3\|CSF2 |
| Adipocytokine signaling pathway | 0.005322 | RXRB\|ACSL6 |
| Ras signaling pathway | 0.005619 | FGF22\|GNB2\|RGL2 |
| Biosynthesis of amino acids | 0.00623 | TKTL1\|ASS1 |
| Amoebiasis | 0.009722 | CSF2\|ITGB2 |
| Glycerophospholipid metabolism | 0.010109 | CDIPT\|TAZ |
| Aldosterone synthesis and secretion | 0.010305 | CYP21A2\|ATF6B |
| Cytokine-cytokine receptor interaction | 0.010642 | IL3\|CSF2\|EPO |
| MAPK signaling pathway | 0.010739 | DAXX\|FLNA\|FGF22 |
| Parathyroid hormone synthesis, secretion and action | 0.011936 | RXRB\|ATF6B |
| Cholinergic synapse | 0.013228 | GNB2\|KCNQ2 |
| TNF signaling pathway | 0.013228 | CSF2\|ATF6B |
| Thyroid hormone signaling pathway | 0.014809 | RXRB\|SLC9A1 |
| Relaxin signaling pathway | 0.017449 | ATF6B\|GNB2 |
| Natural killer cell mediated cytotoxicity | 0.017698 | CSF2\|ITGB2 |
| Dopaminergic synapse | 0.017698 | ATF6B\|GNB2 |
| Oxidative phosphorylation | 0.018201 | ATP6AP1\|NDUFA1 |
| Apelin signaling pathway | 0.019225 | GNB2\|SLC9A1 |
| Retrograde endocannabinoid signaling | 0.022162 | NDUFA1\|GNB2 |
| Gastric cancer | 0.022438 | FGF22\|RXRB |
| Adrenergic signaling in cardiomyocytes | 0.022438 | ATF6B\|SLC9A1 |
| Cushing syndrome | 0.024122 | CYP21A2\|ATF6B |
| Glycosphingolipid biosynthesis - ganglio series | 0.024152 | B3GALT4 |
| Hepatitis B | 0.026446 | ATP6AP1\|ATF6B |
| Selenocompound metabolism | 0.02713 | KYAT1 |
| Alcoholism | 0.03167 | ATF6B\|GNB2 |
| Arginine biosynthesis | 0.03306 | ASS1 |
| Kaposi sarcoma-associated herpesvirus infection | 0.033603 | CSF2\|GNB2 |
| Focal adhesion | 0.037946 | FLNA\|TNXB |
| Proteoglycans in cancer | 0.039323 | FLNA\|SLC9A1 |
| Human immunodeficiency virus 1 infection | 0.042491 | TAPBP\|GNB2 |
| cAMP signaling pathway | 0.043207 | HCN2\|SLC9A1 |
| Pentose phosphate pathway | 0.046273 | TKTL1 |
| Thermogenesis | 0.049479 | ACSL6\|NDUFA1 |
| Alanine, aspartate and glutamate metabolism | 0.054983 | ASS1 |
| Thyroid cancer | 0.056427 | RXRB |
| Ferroptosis | 0.060745 | ACSL6 |
| Tryptophan metabolism | 0.063614 | KYAT1 |
| Valine, leucine and isoleucine degradation | 0.072168 | ACADM |
| Malaria | 0.073586 | ITGB2 |
| Cocaine addiction | 0.073586 | ATF6B |
| Cysteine and methionine metabolism | 0.073586 | KYAT1 |
| Vibrio cholerae infection | 0.075002 | ATP6AP1 |
| Amyotrophic lateral sclerosis (ALS) | 0.076416 | DAXX |
| Pathogenic Escherichia coli infection | 0.08205 | ARPC5L |
| Human papillomavirus infection | 0.091519 | ATP6AP1\|TNXB |
| Non-small cell lung cancer | 0.097371 | RXRB |
| Amphetamine addiction | 0.10013 | ATF6B |
| Epithelial cell signaling in Helicobacter pylori infection | 0.10288 | ATP6AP1 |
| Bile secretion | 0.105622 | SLC9A1 |
| Melanoma | 0.105622 | FGF22 |
| p53 signaling pathway | 0.105622 | TP53I3 |
| Thyroid hormone synthesis | 0.108355 | ATF6B |
| Inositol phosphate metabolism | 0.108355 | CDIPT |
| Bacterial invasion of epithelial cells | 0.108355 | ARPC5L |
| Gastric acid secretion | 0.109719 | SLC9A1 |
| Arrhythmogenic right ventricular cardiomyopathy (ARVC) | 0.112441 | EMD |
| RNA degradation | 0.115154 | SKIV2L |
| Chemical carcinogenesis | 0.119208 | KYAT1 |
| Peroxisome | 0.120556 | ACSL6 |
| Cardiac muscle contraction | 0.124586 | SLC9A1 |
| Insulin secretion | 0.124586 | ATF6B |
| ECM-receptor interaction | 0.124586 | TNXB |
| GABAergic synapse | 0.128598 | GNB2 |
| Longevity regulating pathway | 0.128598 | ATF6B |
| Protein digestion and absorption | 0.129931 | COL11A2 |
| Salivary secretion | 0.129931 | SLC9A1 |
| Hypertrophic cardiomyopathy (HCM) | 0.129931 | EMD |
| Morphine addiction | 0.131262 | GNB2 |
| mRNA surveillance pathway | 0.131262 | UPF3B |
| IL-17 signaling pathway | 0.133919 | CSF2 |
| Small cell lung cancer | 0.133919 | RXRB |
| Fc gamma R-mediated phagocytosis | 0.135244 | ARPC5L |
| Dilated cardiomyopathy (DCM) | 0.137889 | EMD |
| Circadian entrainment | 0.139208 | GNB2 |
| Pancreatic secretion | 0.140526 | SLC9A1 |
| Phosphatidylinositol signaling system | 0.141841 | CDIPT |
| T cell receptor signaling pathway | 0.147083 | CSF2 |
| Ribosome biogenesis in eukaryotes | 0.149692 | POP7 |
| HIF-1 signaling pathway | 0.154886 | EPO |
| Leukocyte transendothelial migration | 0.158761 | ITGB2 |
| Glutamatergic synapse | 0.161335 | GNB2 |
| Serotonergic synapse | 0.162619 | GNB2 |
| Carbon metabolism | 0.165182 | TKTL1 |
| Yersinia infection | 0.170283 | PKN3 |
| Lysosome | 0.172822 | ATP6AP1 |
| Spliceosome | 0.187898 | SF3B6 |
| Ubiquitin mediated proteolysis | 0.190384 | CDC34 |
| Estrogen signaling pathway | 0.191624 | ATF6B |
| Fluid shear stress and atherosclerosis | 0.192863 | ASS1 |
| Parkinson disease | 0.196567 | NDUFA1 |
| Breast cancer | 0.202703 | FGF22 |
| Non-alcoholic fatty liver disease (NAFLD) | 0.205145 | NDUFA1 |
| Hippo signaling pathway | 0.211217 | ITGB2 |
| Protein processing in endoplasmic reticulum | 0.224415 | ATF6B |
| cGMP-PKG signaling pathway | 0.226791 | ATF6B |
| Tight junction | 0.230342 | MYH10 |
| Alzheimer disease | 0.231522 | NDUFA1 |
| Axon guidance | 0.243225 | PLXNA3 |
| Chemokine signaling pathway | 0.253608 | GNB2 |
| Huntington disease | 0.257038 | NDUFA1 |
| Viral carcinogenesis | 0.266108 | ATF6B |
| Endocytosis | 0.313028 | ARPC5L |
| MicroRNAs in cancer | 0.368762 | TNXB |
