# Supplementary material for: Identification of Key LncRNAs and Pathways in Prediabetes and Type 2 Diabetes Mellitus for Hypertriglyceridemia Patients Based on Weighted Gene Co-Expression Network Analysis
Source: Front Endocrinol (Lausanne). 2022 Jan 24;12:800123. doi: 10.3389/fendo.2021.800123 (PMC8818867; doi:10.3389/fendo.2021.800123)
Supplement: Supplementary file 9 [file Table_5.docx]

Table S5 Pathway of Each Gene Participation

| lncRNA ID | Gene | Pathway |
| --- | --- | --- |
| ENST00000503273 | HK3 | Glycolysis / Gluconeogenesis |
|  |  | Metabolic pathways |
|  |  | Neomycin, kanamycin, and gentamicin biosynthesis |
|  |  | HIF-1 signaling pathway |
|  |  | Carbon metabolism |
|  |  | Galactose metabolism |
|  |  | Fructose and mannose metabolism |
|  |  | Starch and sucrose metabolism |
|  |  | Carbohydrate digestion and absorption |
|  |  | Type II diabetes mellitus |
|  |  | Amino sugar and nucleotide sugar metabolism |
|  |  | Central carbon metabolism in cancer |
|  |  | Insulin signaling pathway |
| ENST00000462720  ENST00000480633 | PRKCE | MicroRNAs in cancer |
|  |  | Type II diabetes mellitus |
|  |  | Fc gamma R-mediated phagocytosis |
|  |  | Aldosterone synthesis and secretion |
|  |  | Inflammatory mediator regulation of TRP channels |
|  |  | AGE-RAGE signaling pathway in diabetic complications |
|  |  | Insulin resistance |
|  |  | Sphingolipid signaling pathway |
|  |  | Vascular smooth muscle contraction |
|  |  | Apelin signaling pathway |
|  |  | cGMP-PKG signaling pathway |
|  |  | Tight junction |
| ENST00000485392 | ATF6B | Human T-cell leukemia virus 1 infection |
|  |  | PI3K-Akt signaling pathway |
|  |  | Cortisol synthesis and secretion |
|  |  | Human cytomegalovirus infection |
|  |  | Aldosterone synthesis and secretion |
|  |  | Parathyroid hormone synthesis, secretion and action |
|  |  | TNF signaling pathway |
|  |  | Relaxin signaling pathway |
|  |  | Dopaminergic synapse |
|  |  | Adrenergic signaling in cardiomyocytes |
|  |  | Cushing syndrome |
|  |  | Hepatitis B |
|  |  | Alcoholism |
|  |  | Cocaine addiction |
|  |  | Amphetamine addiction |
|  |  | Thyroid hormone synthesis  Insulin secretion |
|  |  | Longevity regulating pathway |
|  |  | Estrogen signaling pathway |
|  |  | Protein processing in endoplasmic reticulum |
|  |  | cGMP-PKG signaling pathway |
|  |  | Viral carcinogenesis |
