# Supplementary material for: Identification of Key LncRNAs and Pathways in Prediabetes and Type 2 Diabetes Mellitus for Hypertriglyceridemia Patients Based on Weighted Gene Co-Expression Network Analysis
Source: Front Endocrinol (Lausanne). 2022 Jan 24;12:800123. doi: 10.3389/fendo.2021.800123 (PMC8818867; doi:10.3389/fendo.2021.800123)
Supplement: Supplementary file 10 [file Table_6.docx]

Table S6 Validation of Genes via GSE130991

| lncRNA_ID | lncRNA_Gene_Symbol | *P* | Log2FoldChange |
| --- | --- | --- | --- |
| ENST00000503273 | UIMC1 | 0.10 | -0.02 |
| ENST00000462720 | PRKCE | 0.04 | 0.12 |
| ENST00000480633 |  |  |  |
